# Supplementary material for: MetaRibo-Seq measures translation in microbiomes
Source: Nat Commun. 2020 Jun 29;11:3268. doi: 10.1038/s41467-020-17081-z (PMC7324362; doi:10.1038/s41467-020-17081-z)
Supplement: Supplementary file 10 — Supplementary Data 7 [file 41467_2020_17081_MOESM10_ESM.zip › File2/Confidence_High_Taxonomy/415364_out.krona.html]

Javascript must be enabled to view this page.

members
magnitude
magnitudeUnassigned
count
unassigned
taxon
rank

415364\_out

51

superkingdom
51
2

51
1239
phylum

186801
51
class

order
51
186802

186803
51
family

1
genus

SRS144714\_contig\_number\_27325
51
841

species

SRS011084\_contig\_number\_32249SRS013158\_contig\_number\_4801SRS014287\_contig\_number\_18339SRS014613\_contig\_number\_4681SRS014855\_contig\_number\_1071SRS014923\_contig\_number\_41387SRS015065\_contig\_number\_28939SRS015190\_contig\_number\_contig-100\_187.89460SRS015264\_contig\_number\_11368SRS015578\_contig\_number\_33212SRS015579\_contig\_number\_2900SRS015854\_contig\_number\_23205SRS015960\_contig\_number\_20686SRS018984\_contig\_number\_19575SRS019068\_contig\_number\_48054SRS019397\_contig\_number\_10634SRS022137\_contig\_number\_12729SRS024435\_contig\_number\_14137SRS024549\_contig\_number\_3559SRS043001\_contig\_number\_8681SRS043701\_contig\_number\_17079SRS045739\_contig\_number\_contig-100\_896.56000SRS046068\_contig\_number\_contig-100\_350.351SRS050422\_contig\_number\_31692SRS050925\_contig\_number\_6219SRS050941\_contig\_number\_12712SRS052027\_contig\_number\_13832SRS053335\_contig\_number\_17157SRS054956\_contig\_number\_14434SRS055017\_contig\_number\_26728SRS056273\_contig\_number\_29446SRS056519\_contig\_number\_3279SRS058070\_contig\_number\_3431SRS075716\_contig\_number\_1903SRS098061\_contig\_number\_11259SRS098827\_contig\_number\_10450SRS098881\_contig\_number\_4736SRS098881\_contig\_number\_17937SRS1041130\_contig\_number\_3399SRS104485\_contig\_number\_4180SRS105153\_contig\_number\_20050SRS1055034\_contig\_number\_8513SRS142503\_contig\_number\_18968SRS143070\_contig\_number\_16326SRS143598\_contig\_number\_19957SRS143780\_contig\_number\_17450SRS147919\_contig\_number\_15547SRS148159\_contig\_number\_38115SRS149879\_contig\_number\_24936SRS893378\_contig\_number\_11456
166486
50
